# A cross-sectional study of factors associated with unstable housing among marginalized people who use drugs in Ottawa, Canada

**DOI:** 10.1371/journal.pone.0253923

**Published:** 2021-07-01

**Authors:** Ellen C. Rowlands Snyder, Lisa M. Boucher, Ahmed M. Bayoumi, Alana Martin, Zack Marshall, Rob Boyd, Sean LeBlanc, Mark Tyndall, Claire E. Kendall

**Affiliations:** 1 School of Epidemiology and Public Health, University of Ottawa, Ottawa, Ontario, Canada; 2 MAP Centre for Urban Health Solutions, Li Ka Shing Knowledge Institute, St. Michael’s Hospital, Toronto, Ontario, Canada; 3 Division of General Internal Medicine, St. Michael’s Hospital, Toronto, Ontario, Canada; 4 Department of Medicine, University of Toronto, Toronto, Ontario, Canada; 5 Institute of Health Policy, Management, and Evaluation, University of Toronto, Toronto, Ontario, Canada; 6 Somerset West Community Health Centre, Ottawa, Ontario, Canada; 7 PROUD Community Advisory Committee, Ottawa, Ontario, Canada; 8 School of Social Work, McGill University, Montreal, Quebec, Canada; 9 Sandy Hill Community Health Centre, Ottawa, Ontario, Canada; 10 Drug Users Advocacy League, Ottawa, Ontario, Canada; 11 Faculty of Medicine, School of Population and Public Health, University of British Columbia, Vancouver, British Columbia, Canada; 12 C.T. Lamont Primary Health Care Research Centre, Bruyère Research Institute, Ottawa, Ontario, Canada; New York City Department of Health and Mental Hygiene, UNITED STATES

## Abstract

**Introduction:**

Housing affects an individual’s physical and mental health, particularly among people who use substances. Understanding the association between individual characteristics and housing status can inform housing policy and help optimize the care of people who use drugs. The objective of this study was to explore the factors associated with unstable housing among people who use drugs in Ottawa.

**Methods:**

This is a cross-sectional analysis of data from 782 participants in the Participatory Research in Ottawa: Understanding Drugs (PROUD) Study. PROUD is a prospective cohort study of people who use drugs in Ottawa. Between March and December 2013, participants were recruited through peer-based recruitment on the streets and in social services settings and completed a peer-administered questionnaire that explored socio-demographic information, drug use patterns, community integration, experiences with police and incarceration, and access to health care and harm reduction services. Eligibility criteria included age of 16 years or older, self-reported illicit drug use within the past 12 months and having lived in Ottawa for at least 3 months. Housing status was determined by self-report. “Stable housing” was defined as residence in a house or apartment and “unstable housing” was defined as all other residence types. Exploratory multivariable logistic regression analyses of the association between characteristics of people who use drugs and their housing status were conducted.

**Results:**

Factors that were associated with unstable housing included: recent incarceration; not having a regular doctor; not having received support from a peer worker; low monthly income; income source other than public disability support payments; and younger age. Gender, language, ethnicity, education level, opioid use and injection drug use were not independently associated with housing status.

**Conclusions:**

People who use drugs face significant barriers to stable housing. These results highlight key areas to address in order to improve housing stability among this community.

## Introduction

Housing instability is associated with an increased risk of homelessness [1] and is itself known to have both a direct and indirect impact on physical and mental health [2]. Unstable housing is associated with increased use of health and social services, poorer health outcomes, and increased mortality, making it a significant public health issue [3–6]. Given the importance of housing status as a determinant of health, studies have identified predictors of unstable housing among the general population, including male gender [5], younger age [4], mental illness (depression and psychiatric hospitalization), having a lower income [6,7], having less than a high school education [6], and being unemployed [4,8]. Recent incarceration has been associated with loss of housing and greater length of time unstably housed [4,9]. A rating of “severe” on the Drug Abuse Screening Test has been associated with a higher risk of housing instability [5] and greater difficulty in obtaining stable housing [10]. Among people who use drugs, unstable housing has also been associated with higher risk drug use [11–16]. It is thus unsurprising that unstable housing has been found to increase hospitalization and health care costs among people who use drugs [17].

While the impacts of housing status on drug use and vice-versa have been studied, there is a significant gap in our knowledge of factors associated with unstable housing specifically among people who use drugs. Identifying such factors is essential to effectively target housing interventions and resources for this population. In order to better understand these issues, we studied the factors associated with unstable housing among marginalized people actively using illicit drugs in Ottawa, Ontario, Canada. Using a community-led approach, we used data from the Participatory Research in Ottawa: Understanding Drugs (PROUD) Study [18], a community-based cohort of people who use drugs in Ottawa, to examine demographic, economic and social variables impacting the lives of participants.

## Methods

### Study setting

Ottawa, Ontario is a city with approximately one million residents, and is the capital of Canada. Ontario has a single payer publicly funded health system with universal access for necessary physician services.

### Study design

The PROUD Study is a cross-sectional cohort study examining HIV risk among people who use drugs in Ottawa, Ontario and has been described in detail elsewhere [18]. PROUD incorporates community-based participatory research principles of community engagement, trust and ownership by actively engaging a Community Advisory Committee (CAC) consisting of people with drug use experience and their allies [19–21]. The survey was created by the CAC to be relevant for the local community of marginalized people who use drugs. It includes questions in eight broad sections chosen by the CAC to describe the HIV risk environment in their community. These sections include: characteristics of drug users in Ottawa; drug use patterns; access to harm reduction services; health status and health care access; sexual activities and history; connections to community; housing and homelessness; and the influence of law enforcement. Between March and December 2013, participants were recruited through peer-based recruitment on the streets and in social services settings frequented by people who use drugs in Ottawa. Eligibility criteria included age 16 years or older, self-reported drug use within the past 12 months (not including exclusive marijuana use) and having lived in Ottawa for at least 3 months. Participants completed a one-time peer or medical student administered quantitative survey and received a cash honorarium of $20.

This study received approval from the Ottawa Health Sciences Network Research Ethics Board (OHSN-REB #20120566-01H) and the Ottawa Public Health Research Ethics Board. Consent to publish was obtained as part of informed consent. Written consent for this study was obtained prior to completion of the survey.

#### Outcome

Our main outcome variable, unstable housing, was based on self-reported type of residence in response to the question “where are you living right now?” Participants were asked to select from the following list: Own apartment/house, rooming house, shelter, supportive housing, recovery house/detox, friends/relatives house/place, street/homeless, hotel/motel room, other. In consultation with the CAC, we defined “stable housing” as residence in “own apartment/house” and “unstable housing” as all other residence types. This definition was chosen by the community to capture a measure of permanent housing that supports autonomy and is consistent with that used in previous Canadian studies [22].

#### Variables of interest

To identify factors associated with unstable housing, we considered a number of explanatory variables including self-reported sociodemographic factors: gender (male vs. female vs. other), age (<25 years, 25–34 years, 35–44 years or 45+ years), and ethnicity (Aboriginal vs. Caucasian vs. neither). Gender was identified as male, female, or "other" if the participant reported trans male, trans female, Two-Spirited, other, or no answer. Given their demographic importance, we excluded any participants with missing responses for age and gender.

Drug use variables included opioid use in the last 12 months (yes vs. no) and injection drug use in the last 12 months (yes vs. no). Opioid use was categorized as "yes" if the participant reported using any of the following when asked about each drug in turn: heroin, oxycodone, morphine, percocet, fentanyl, or hydromorphone.

Socioeconomic variables included self-reported education level (less than high school vs. high school graduate vs. some college/university vs. college/university graduate), estimated monthly income (<$499 vs. $500–999 vs. $1000–1999 vs. >$2000), and source of income (disability payments (Ontario Disability Support Program [ODSP]) vs. income assistance (Ontario Works) vs. neither).

The following variables were dichotomized as “yes” if the participant responded in the affirmative and “other” if the participant responded “no”, “no answer”, “don’t know”: spent overnight or longer in jail in the last 12 months; currently on methadone; has a regular doctor; ever received support from a peer worker. A peer worker is a non-professional worker who has lived experience with drug use and plays a supportive or navigator role in accessing health or social services. If responses were missing due to the skip pattern in the survey, these were recoded as “no”. Complete case analysis for the model was performed by removing all responses other than “yes” and “no” from dichotomous variables and the results were unchanged. Results with low cell counts are reported as a range in order to preserve the confidentiality of the participants.

#### Analyses

We used descriptive statistics to describe the study population and to compare those who had stable and unstable housing. We used logistic regression to examine which participant characteristics were associated with unstable housing. We explored covariates that were chosen a priori by the research team, including the CAC, because they were felt to be potential independent predictors or confounders of housing status. The relationship between the following variables was examined for the presence of collinearity by examining both the variance inflation factor and the correlation coefficient: income; income source; education; access to a regular doctor; methadone treatment. None of these variables displayed a high degree of collinearity (defined as a variance inflation factor >10 or a correlation coefficient of >0.7) and therefore all were included in the model. We report associations as odds ratios from univariable and multivariable analyses with 95% confidence intervals. We determined a priori that statistically significant proportional differences would be at the p< = 0.05 level. All analyses were conducted using SPSS version 24.

## Results

**Fig 1:**
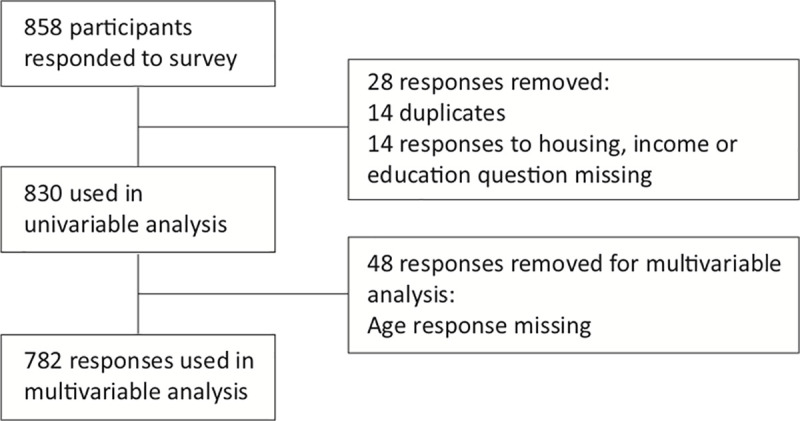
STROBE diagram.

There were 858 responses to the PROUD survey between March and December 2013. For this analysis, 28 survey responses were eliminated before univariable analysis was conducted: 14 as duplicates and 14 because the participant had not responded to key questions required for analysis of housing status. We also excluded 48 participants who had missing age responses, leaving 782 participants for analysis (see Fig 1). Of the remaining 782 participants, 503 (60.6%) reported unstable housing (Table 1). Participants were predominantly male (n = 618; 74.5%), the mean age was 41.8 years, 586 (70.6%) self-identified as Caucasian, 393 (47.3%) reported injecting drugs within the last 12 months, and 528 (63.6%) reported using opioids within the past 12 months.

**Table 1:** Characteristics of participants stratified by housing status (n = 830).

		Total % (n)	Housing status		
			Stable (n = 327) % (n)	Unstable (n = 503) % (n)	p-value
**Age (years)**	<25	6.9 (57)	4.9 (16)	8.2 (41)	
	25–34	18.8 (156)	12.2 (40)	23.1 (116)	
	35–44	25.5 (212)	25.1 (82)	25.8 (130)	
	45+	43.0 (357)	51.4 (168)	37.6 (189)	<0.001
	Missing	5.8(48)	6.4 (21)	5.4 (27)	
**Gender**	Male	74.5 (618)	70.6 (231)	76.9 (387)	0.103
	Female	20–30 (166–249)	25–30 (82–98)	20–25 (101–126)	
	Other	0–5 (0–42)	0–5 (0–16)	0–5 (0–25)	
**First Language**	English	75.8 (629)	78.6 (257)	74.0 (372)	0.299
	French	16.3 (135)	14.7 (48)	17.3 (87)	
	Other	8.0 (66)	6.7 (22)	8.7 (44)	
**Ethnicity**	Aboriginal	18.2 (151)	18.7 (61)	17.9 (90)	0.327
	Caucasian	70.6 (586)	72.2 (236)	69.6 (350)	
	Other	11.2 (93)	9.2 (30)	12.5 (63)	
**Injected drugs in the last 12 months**	Yes	47.3 (393)	51.1 (167)	44.9 (226)	0.083
	Other	52.7 (437)	48.9 (160)	55.1 (277)	
**Opioid use in the last 12 months**	Yes	63.6 (528)	65.7 (215)	62.2 (313)	0.303
	Other	36.4 (302)	34.3 (112)	37.8 (190)	
**Education level**	College/university graduate	10.1 (84)	11.0 (36)	9.5 (48)	
	Some college/university	14.1 (117)	16.2 (53)	12.7 (64)	
	High school graduate	28.2 (234)	31.2 (102)	26.2 (132)	
	Some high school or less	47.6 (395)	41.6 (136)	51.5 (259)	0.047
**Monthly income**	>$2000	13.7 (114)	16.2 (53)	12.1 (61)	
	$1000–1999	33.0 (274)	39.4 (129)	28.8 (145)	
	$500–999	36.7 (305)	36.1 (118)	37.2 (187)	
	Less than $499	16.5 (137)	8.3 (27)	21.9 (110)	<0.001
**Income source**	ODSP	39.2 (325)	52.0 (170)	30.8 (155)	<0.001
	Ontario Works	37.7 (313)	27.5 (90)	44.3 (223)	
	Neither	23.1 (192)	20.5 (67)	24.9 (125)	
**In jail in the last 12 months**	Yes	37.0 (307)	26.6 (87)	43.7 (220)	<0.001
	Other	63.0 (523)	73.4 (240)	56.3 (283)	
**Currently on Methadone**	Yes	23.3 (193)	30.0 (98)	18.9 (95)	<0.001
	Other	76.7 (637)	70.0 (229)	81.1 (408)	
**Has regular doctor**	Yes	55.2 (458)	66.1 (216)	48.1 (242)	<0.001
	Other	44.8 (372)	33.9 (111)	51.9 (261)	
**Ever received support from a peer worker**	Yes	39.9 (331)	45.0 (147)	36.6 (184)	0.016
	Other	60.1 (499)	55.0 (180)	63.4 (319)	

The characteristics of the study sample stratified by housing status are presented in Table 1. Age differed among those with stable versus unstable housing, with more participants with unstable housing falling into younger age groups. For instance, 24.4% of individuals with unstable housing were 25–34 years old, while only 13.1% of those with stable housing fell into this age category. Unsurprisingly, a larger proportion of participants with unstable housing reported less than a high school education, an income of less than $499 per month from all sources, and income assistance among their sources of income. Among participants with unstable housing, the proportion reporting incarceration within the last 12 months was also higher than among those with stable housing. While the majority of participants overall did not report currently taking methadone, among those with unstable housing fewer reported current methadone use than among those with stable housing. More participants with unstable housing also reported not having a regular doctor.

The results of the univariable and multivariable analyses are shown in Table 2. In univariable analyses, unstable housing status was associated with younger age, monthly income below $499, not receiving disability payments, incarceration within the last 12 months, no current methadone use, not having a regular doctor, and not receiving support from a peer worker. In multivariable analyses, unstable housing remained associated with all of these variables except for no current methadone use.

**Table 2:** Unadjusted and adjusted analysis of characteristics associated with unstable housing, adjusted for listed characteristics (n = 782).

		Univariable analysis		Multivariable analysis	
		Unadjusted odds ratio	p-value	Adjusted odds ratio	p-value
**Age**	<25	**2.28 (1.23–4.21)**	**0.009**	1.78 (0.90–3.52)	0.095
	25–34	**2.58 (1.70–3.90)**	**<0.001**	**2.21 (1.38–3.52)**	**0.001**
	35–44	1.41 (1.00–1.99)	0.052	1.30 (0.88–1.90)	0.184
	45+	ref		ref	
**Gender**	Male	ref		ref	
	Female	0.74 (0.53–1.01)	0.060	0.79 (0.54–1.15)	0.215
	Other	0.48 (0.13–1.80)	0.274	0.24 (0.05–1.16)	0.075
**First Language**	English	ref		ref	
	French	1.25 (0.85–1.84)	0.254	1.30 (0.84–2.01)	0.244
	Other	1.38 (0.81–2.36)	0.237	1.04 (0.52–1.95)	0.991
**Ethnicity**	Aboriginal	ref		ref	
	Caucasian	1.01 (0.70–1.45)	0.978	1.28 (0.83–1.97)	0.272
	Other	1.42 (0.83–2.45)	0.202	1.40 (0.74–2.64)	0.302
**Injected drugs in the last 12 months**	Other	ref		ref	
	Yes	0.78 (0.59–1.03)	0.084	0.92 (0.62–1.37)	0.692
**Opioid use in the last 12 months**	Other	ref		ref	
	Yes	0.86 (0.64–1.15)	0.303	0.93 (0.62–1.38)	0.709
**Education level**	College/university graduate	ref		ref	
	Some college/university High school graduate	0.91 (0.52–1.59)	0.731	1.01 (0.53–1.94)	0.977
		0.97 (0.59–1.61)	0.907	0.86 (0.48–1.55)	0.620
	Some high school or less	1.43 (0.88–2.31)	0.145	1.29 (0.73–2.26)	0.380
**Monthly income**	>2000	ref		ref	
	$1000–1999	0.98 (0.63–1.51)	0.916	1.64 (0.98–2.74)	0.060
	$500–999	1.38 (0.89–2.13)	0.149	**2.04 (1.23–3.39)**	**0.006**
	**$Less than 499**	**3.54 (2.02–6.19)**	**<0.001**	**4.90 (2.56–9.39)**	**<0.001**
**Income source**	ODSP	ref		ref	0.043
	**Ontario Works (no ODSP)**	**2.72 (1.96–3.77)**	**<0.001**	**1.53 (1.02–2.28)**	**0.038**
	**Neither**	**2.05 (1.42–2.96)**	**<0.001**	**1.59 (1.04–2.41)**	**0.031**
**In jail in the last 12 months**	Other	ref		ref	
	**Yes**	**2.15 (1.59–2.90)**	**<0.001**	**2.22 (1.57–3.15)**	**<0.001**
**Currently on Methadone**	Yes	ref		ref	
	Other	**1.84 (1.33–2.55)**	**<0.001**	1.49 (1.00–2.24)	0.053
**Has regular doctor**	Yes	ref		ref	
	Other	**2.10 (1.57–2.80)**	**<0.001**	**1.67 (1.20–2.34)**	**0.003**
**Ever received support from a peer worker**	Yes	ref		ref	
	Other	**1.42 (1.07–1.88)**	**0.016**	**1.40 (1.01–1.94)**	**0.041**

After adjustment, individuals aged 25–34 years had higher odds of reporting unstable housing compared with those 45 years and older (adjusted odds ratio [AOR] 2.21, 95% confidence interval [95%CI]: 1.38–3.52). Income showed a strong association with housing stability: Lower monthly income was associated with unstable housing (<$499: AOR 4.90, 95%CI 2.56–9.39; $500–999: AOR 2.04, 95%CI 1.23–3.39) when compared to an income of >$2000. Source of income was also associated with unstable housing, with increased odds of unstable housing among those receiving no financial support or receiving only income assistance relative to those receiving disability payments (no financial support: AOR 1.59, 95%CI 1.04–2.41; income assistance AOR 1.53, 95%CI 1.02–2.28). Other factors that remained associated with increased odds of unstable housing include incarceration within the last 12 months (AOR 2.22, 95%CI 1.57–3.15), not having a regular doctor (AOR 1.67, 95%CI 1.20–2.34), and not having received support from a peer worker (AOR 1.40, 95%CI 1.01–1.94).

## Discussion

We conducted a study exploring factors associated with housing stability among people who use drugs in Ottawa. In our study, 61% of participants reported unstable housing. We found that lower age, lower income, not receiving disability support payments, recent incarceration, no access to a regular doctor, and not receiving services from a peer worker were all independently associated with unstable housing status.

Our findings on the association between having a doctor or peer worker support and housing stability suggest the importance of care models [23,24] that emphasize improved coordination and integration of medical, mental health, and substance use care for people who use drugs [25,26]. Although we did not find a significant association between current methadone use and housing stability, access to addictions treatment has been associated with stable housing in previous studies [27,28]. Within an integrated, team-based model of care, peer workers are increasingly included as part of health and social services delivery. Peer workers are thought to meet the needs of marginalized groups through shared lived-experience and strengths-focused social and practical support [29,30]. However, there is limited high quality research examining the impact of peer workers on housing stability among people who use drugs [31]. The association between peer support and housing is a novel and potentially important finding that should be further explored through longitudinal studies in order to understand the impact of including peer workers within health and social services programs, including housing services.

It is well established that drug use is prevalent among people with unstable housing [11,12] and that high intensity drug use is associated with unstable housing [5,10]. It was therefore surprising that, in our study, injection drug use and use of opioids were not associated with unstable housing. Polysubstance use is complex and it is possible that, among this cohort of marginalized people who actively use drugs, type of drug use may not be as salient a distinction as it is among a broader population of people at risk of unstable housing. While input from community members with lived experience of drug use was sought to assist in identifying the most relevant variables to include, it is possible that our data regarding type and route of drug use was insufficient to fully capture associations between overall patterns of drug use and housing status.

We also found a clear association between lower self-reported income and increasing odds of unstable housing among people who use drugs, which is consistent with the literature [6,7]. In our study each lower income category had a higher odds of unstable housing, with almost 5 times the odds among those reporting less than $499 per month. Source of income was also found to be independently associated with unstable housing. Compared to disability payments, both income assistance and other forms of reported income were associated with unstable housing. Income assistance benefits are lower and less stable than disability payments in Ontario, which could contribute to housing instability through decreased overall income [32]. These findings could inform programs aiming to address poverty and highlight the fact that people who use drugs may be a priority population for such programs.

Incarceration within the previous year more than doubled the odds of unstable housing in our study population. This is consistent with previous literature showing incarceration to be associated with loss of stable housing and greater difficulty establishing stable housing [4,9,33]. A recent study among people who use drugs and who were incarcerated also showed that certain types of housing are associated with a greater chance of resuming substance use following incarceration [34]. Previous studies using data from the PROUD cohort have also found incarceration to be associated with increased emergency department visits [35]. This speaks to the wide-reaching impact of interactions with the criminal justice system on the lives of people who use drugs. Our findings indicate that individuals who have recently been incarcerated may represent a sub-population that is particularly vulnerable to housing instability and could benefit from additional supports to mitigate some of the unintended consequences of incarceration that perpetuate cycles of marginalization.

### Strengths and limitations

The strengths of this study include the community-based participatory design that engaged community members with lived drug use experience in decision-making at every step of the research process. This drew on a “Nothing about us without us” approach, anchored in the Greater Meaningful Involvement of People with HIV (GIPA-MIPA) principles [20]. This approach allowed for the collection of rich survey data on a typically hard to reach, marginalized population. It was the largest study of its kind in Ottawa at the time and one of the largest cohorts of people who use drugs nationally [18].

Our study also has several limitations. First, our street-based sampling methods recruited a non-random sample of a highly disadvantaged population. Therefore, the findings may not be generalizable to the entire population of people who use drugs. Second, the face-to-face interviews used to elicit self-reported information on stigmatized and illegal activities could have contributed to social desirability bias as well as recall bias. However, this was mitigated by the use of trained peer researchers to conduct the interviews. Third, our results could be impacted by unmeasured confounding effects, especially given the complex nature of the risk environment for people who use drugs [36].

Finally, based on community input we used a specific definition of housing stability that emphasized permanent housing and autonomy, which may not reflect other definitions, including for different jurisdictions or populations. Those types of housing that are included in our definition of unstable housing due to their temporary nature, such as supportive housing and recovery housing make up a very small portion of those who reported “unstable housing” and are therefore unlikely to influence our results. Although the CAC considered that the housing we categorized as “stable” was more likely to be permanent, our definition was based on the self-reported place of residence at a single moment in time in our study. While this definition has been used in other studies [22], it may not fully reflect the fluidity of housing situations for people who use drugs [4]. As such, our findings should be interpreted carefully in the context of our community-based definition of housing stability and data limitations.

While our results add to the body of knowledge on housing and drug use, given the cross-sectional nature of this study, we can only identify factors associated with unstable housing and cannot establish causality. Longitudinal studies in this area are needed to explore the temporal relationship between the variables that we identified and housing stability.

### Conclusions

Unstable housing has a significant impact on health. While factors associated with unstable housing have been well established, few studies have examined characteristics specific to people who use drugs. Our study identifies variables associated with risk of unstable housing among a marginalized group of people who use drugs in Ottawa. The findings suggest that existing income support programs are insufficient to address pervasive precarious housing among people who use drugs, as are post-incarceration support programs. Access to a regular physician and support from a peer worker may also play a role in housing stability, which speaks to the value of integrated services targeting the health needs of this community [37,38].
